# Association between fatigue and self-care in patients with heart failure: a cross-sectional study

**DOI:** 10.1590/1980-220X-REEUSP-2025-0493en

**Published:** 2026-09-25

**Authors:** Weslley Italo Ferreira de Oliveira Silva, Yzis Oliveira Pontes Pereira, Filipe Matheus Correia Vieira, Fernanda Kelly Oliveira de Albuquerque, Ricardo Costa da Silva, José Claudio Garcia Lira, Mailson Marques de Sousa

**Affiliations:** 1 Universidade Federal da Paraíba, Departamento de Enfermagem Clínica, Programa de Pós-Graduação em Enfermagem, João Pessoa, PB, Brazil.; 2 Universidade Estadual de Goiás, Departamento de Enfermagem, Ceres, GO, Brazil.; 3 Universidade Federal do Piauí, Departamento de Enfermagem, Programa de Pós-Graduação em Saúde e Comunidade, Floriano, PI, Brazil.

**Keywords:** Self-Care, Fatigue, Heart Failure, Cross-Sectional Studies

## Abstract

**Objective:**

To analyze the association between fatigue, self-care, and sociodemographic and clinical variables in patients with heart failure.

**Method:**

A cross-sectional and correlational study, conducted at a university hospital in the Northeast region of Brazil. The Brazilian versions of the *Dutch Fatigue Scale*, *Dutch Exertion Fatigue Scale,* and the *European Heart Failure Self-care Behavior Scale* were used. The analysis included descriptive statistics and Spearman correlation.

**Results:**

Ninety-nine patients with heart failure participated. Substantial fatigue was observed in 71.7% and fatigue on exertion in 66.7% of the sample. The mean self-care score was 28.41 ± 7.98. There was no significant association between fatigue and self-care. A negative correlation was found between age and exertional and substantial fatigue, and a positive correlation was found between the duration of treatment and age. There was a positive correlation between functional capacity, exertional fatigue, and substantial fatigue.

**Conclusion:**

The findings reinforce the importance of symptom awareness and management in self-care. Health interventions are timely for promoting the quality of life of this population.

## INTRODUCTION

Heart failure (HF) is a clinical syndrome characterized by structural and/or functional changes in the heart that compromise ventricular filling and/or ejection, resulting in inadequate or reduced cardiac output. It is estimated that 64 million people worldwide have HF^(1)^. In Brazil, HF is the leading cause of hospital readmission and high rates of in hospital mortality, resulting in high financial costs and overburdening the Brazilian Public Health System (*SUS*)^(2)^.

In HF patients, one of the main factors associated with clinical decompensation and hospital readmission is poor adherence to self-care behaviors^(3)^. Self-care is defined as a continuous process that involves maintaining health through the adoption of practices that promote well-being and the management of chronic disease through the dimensions of maintenance, monitoring, and management^(4)^.

A recent systematic review demonstrated that individuals with better self-care behaviors have a better quality of life, as well as lower hospitalization and mortality rates^(5)^. However, the occurrence of symptoms throughout life can influence self-care performance in different ways, sometimes acting as a stimulus for acquiring skills, and other times as an inhibitor of the desire to engage in self-care^(4)^.

People with HF often experience persistent symptoms, among which fatigue stands out due to its subjective and debilitating nature, resulting in significant impairment of daily functioning and, especially, self-care behaviors. Although conceptualized in a heterogeneous way in the literature^(6)^, fatigue can be categorized into two distinct forms that can occur simultaneously. General fatigue, unrelated to exertion, and exertional fatigue, manifested during or after physical activity, characterizing exercise intolerance^(5)^.

A European study showed that exertional fatigue was significantly associated with poor adherence to self-care over time, independent of sleep disorders, mood changes, and other clinical conditions^(7)^. Convergingly, a scoping review that analyzed 19 studies identified fatigue as a significant obstacle to self-care practices^(6)^. However, only one of these studies was conducted in Brazil, highlighting the need to expand national research on the topic.

Fatigue compromises self-care behavior by promoting physical and cognitive changes that impair health decision making. Individuals with HF may experience reduced energy, concentration, and memory, hindering planning of daily activities related to pharmacological and non-pharmacological management, including regular medication use, meal preparation, physical activity, and early recognition of signs and symptoms of clinical and social decompensation^(5,7)^.

In the Latin American context, there is still no consolidated evidence exploring the association between fatigue and self-care in patients with HF, which limits the understanding of the phenomenon. Therefore, investigating this interface is essential to identify knowledge gaps, since the findings can support healthcare professionals, especially nurses, in directing interventions for symptom management and therapeutic adherence. Given this context, the objective was to analyze the association between fatigue, self-care, and sociodemographic and clinical variables in patients with heart failure.

## METHOD

### Study Design

This cross-sectional study was conducted in the cardiology outpatient clinic of a public teaching hospital affiliated with the Brazilian Public Health System (*SUS*), located in the city of João Pessoa, Paraíba, Brazil.

### Population, Selection Criteria and Definition of the Sample

The study population consisted of patients with HF who were receiving outpatient follow-up at the aforementioned hospital. Sample size estimation was performed using OpenEpi software, version 3.01, based on a 5% margin of error (error = 0.05), with a 95% confidence level, considering the true proportion to be 50% (p = 0.50) due to the variability in fatigue prevalence in the literature^(5)^. Therefore, the minimum sample size was estimated at 94 participants.

Individuals with a clinical diagnosis of HF and aged 18 years or older were included. Patients classified as NYHA class IV, those diagnosed with cancer, lung disease, acute myocardial infarction within the last three months, those undergoing treatment for anxiety or depression, those using sleep-inducing medications documented in their medical records, as well as those with cognitive impairment that compromised their understanding of the study’s objectives, their response, or their completion of the data collection instruments were excluded. Patients classified in class IV were excluded due to advanced HF symptoms^(2)^. Patients disoriented with regard to time, space, or person were also excluded, a condition assessed through observation by the researchers and through the following questions: ‘What’s your full name?’, ‘How old are you?’, ‘What day is it today?’ and ‘Where are we at this moment?’.

### Data Collection

Data were collected by two duly trained nursing undergraduates between March and November 2024 through individual interviews in a private setting, using three instruments, namely:

For sample characterization, a form^(2)^ was used containing sociodemographic and clinical variables (origin, age in years, sex, self-reported skin color, education level, marital status, family income, occupational status, etiology of HF, time since diagnosis, HF NYHA functional class, left ventricular ejection fraction (LVEF) recorded in a transthoracic echocardiogram report performed in the last six months, associated chronic comorbidities, current medication therapy, and history of emergency hospitalizations.

For fatigue assessment, the translated and adapted Portuguese versions of the *Dutch Fatigue Scale* (DUFS) and *Dutch Exertion Fatigue Scale* (DEFS)^(8)^ were used. The DUFS consists of eight items that measure the frequency of fatigue in the three to six months prior to data collection. This scale assesses aspects such as general fatigue, reduced concentration, decreased motivation or interest, difficulty initiating or maintaining daily activities, and the individual’s perception of the intensity of physical and mental fatigue. Responses follow a five-point Likert scale, ranging from 1 (never) to 5 (always). The total score ranges from 8 to 40, with higher values indicating greater fatigue intensity^(8)^.

The DEFS consists of nine items that assess the frequency of fatigue during the performance of daily activities, such as walking, shopping, or collecting garbage. The responses follow a five-point Likert-type scale, ranging from 1 (activity does not cause fatigue) to 5 (extremely fatiguing activity). The total score can range from 9 to 45 points, with higher values indicating greater fatigue intensity. The scales showed Cronbach’s alpha values of 0.84 for DUFS and 0.92 for DEFS^(8)^.

Brazilian researchers suggest cut-off points for interpreting the scales: ≥ 14.5 on the DUFS to characterize ‘substantial fatigue’ and ≥ 12.5 on the DEFS for ‘substantial exertional fatigue’. It should be noted that lower values should not be interpreted as an absence of fatigue, but rather as less pronounced levels. These parameters were previously used in a national study^(9)^.

Self-care behavior was assessed by the *European Heart Failure Self-care Behavior Scale* (EHFScBS), translated and validated for Brazilian Portuguese^(10)^. The instrument contains 12 items in a single domain, with responses on a Likert scale from 1 (“strongly agree”) to 5 (“strongly disagree”), totaling scores between 12 and 60 points, where higher values indicate worse self-care behavior. The reliability estimate showed a Cronbach’s alpha of 0.70 and an intraclass correlation coefficient of 0.87^(10)^.

### Data Analysis and Processing

Data were organized in Microsoft Excel and analyzed in the software Jamovi, version 2.6. Descriptive statistics (absolute and relative frequencies for categorical variables; mean, standard deviation, median, and interquartile range for numerical variables) and inferential statistics were used. Normality was verified using the Shapiro-Wilk test, which indicated a non parametric distribution. To verify the relationships between sociodemographic, clinical, fatigue, and self-care variables, Spearman’s correlation coefficient was applied. The strength of the correlations was classified as weak (0.1–0.3), moderate (0.4–0.6), or strong (0.7–0.9)^(11)^. A significance level of 5% and a p-value of 0.5% were adopted <0.05.

### Ethical Aspects

The study was approved by the Research Ethics Committee (CEP) of the Lauro Wanderley University Hospital under opinion number 6.704.645. All participants received verbal and written information about the research and confirmed their agreement by signing the Free Informed Consent Form (FICF), in duplicate.

## RESULTS

The study included 99 patients with heart failure, the majority of whom were male (n = 52; 52.5%), mixed race (n = 59; 59.6%), married (n = 54; 54.5%), retired (n = 55; 55.6%), with an income of up to one Brazilian minimum wage (n = 68; 68.7%), and from João Pessoa, PB (n = 66; 66.7%). The average age of the participants was 59.33 (SD = 10.47) and 7.04 (SD = 5.21) years of schooling.

Regarding clinical variables, 74 (74.7%) had heart failure of ischemic etiology, 46 (46.5%) were categorized in NYHA class II, with a mean LVEF of 37.57% (SD = 12.86). High blood pressure was the most prevalent comorbidity associated with HF (n = 67; 67.7%). Approximately 62 (62.6%) engaged in physical activity and 89 (89.9%) reported that they did not consume alcoholic beverages.

Using the DUFS, approximately 71.7% of patients experienced substantial fatigue (≥ 14.5), while in the DEFS, 66.7% experienced substantial fatigue on exertion (≥ 12.5). In turn, self-care was 28.41 (SD = 7.98), according to EHFScBS, as per Table 1.

**Table 1:** Scores from the DUFS, DEFS, and EHFScBS instruments – João Pessoa, PB, Brazil, 2024.

Variables	Mean ± standard deviation	Minimum value	Maximum value	Median
DUFS Score	20.92 ± 8.11	8	40	21.00
DEFS Score	17.99 ± 8.35	9	45	16.00
EHFScBS Score	28.41 ± 7.98	14	48	27.00

Abbreviations: DUFS – *Dutch Fatigue Scale*; DEFS – *Dutch Exertion Fatigue Scale*; EHFScBS – *European Heart Failure Self-care Behavior Scale*.

In Table 2, the items “I weigh myself every day” (item 1), “If I gain 2kg in 1 week, I should contact my doctor or nurse (or some health service)” (item 5), “I limit the amount of liquids I drink (no more than 1.5 to 2 liters per day)” (item 6) and “I exercise regularly” (item 12) presented the highest score, demonstrating low adherence. Items 2, 3, 4, 7, 8, 9, 10, and 11 showed lower scores, which represents better agreement regarding self-care activities.

**Table 2:** Frequency distribution of responses from the self-care scale (EHFScBS) – João Pessoa, PB, Brazil, 2024.

Questions	I completely agree. n (%)	I almost always agree. n (%)	Sometimes I agree. n (%)	I almost never agree. n (%)	I completely disagree. n (%)
1. I weigh myself every day.	8 (8.1)	10 (10.1)	18 (18.2)	12 (12.1)	51 (51.5)
2. If I feel short of breath, I rest.	68 (68.7)	2 (2.0)	5 (5.1)	7 (7.1)	17 (17.2)
3. If my shortness of breath worsens, I contact my doctor or nurse (or some other health service).	62 (62.6)	7 (7.1)	4 (4.0)	10 (10.1)	16 (16.2)
4. If my feet or legs become more swollen than usual, I contact my doctor or nurse (or some other health service).	69 (69.7)	3 (3.0)	1 (1.0)	3 (3.0)	23 (23.2)
5. If I gain 2kg in 1 week, I contact my doctor or nurse (or some other health service).	40 (40.4)	7 (7.1)	3 (3.0)	7 (7.1)	42 (42.4)
6. I limit the amount of liquids I drink (no more than 1.5 to 2 liters per day).	29 (29.3)	7 (7.1)	7 (7.1)	3 (3.0)	53 (53.5)
7. I rest during the day.	79 (79.8)	5 (5.1)	8 (8.1)	1 (1.0)	6 (6.1)
8. If my fatigue increases, I contact my doctor or nurse (or some other health service).	75 (75.8)	7 (7.1)	5 (5.1)	2 (2.0)	10 (10.1)
9. I eat a low-salt diet.	76 (76.8)	5 (5.1)	7 (7.1)	3 (3.0)	8 (8.1)
10. I take my medication as prescribed.	83 (83.8)	6 (6.1)	5 (5.1)	2 (2.0)	3 (3.0)
11. I get the flu vaccine every year.	57 (57.6)	7 (7.1)	3 (3.0)	5 (5.1)	27 (27.3)
12. I exercise regularly.	26 (26.3)	10 (10.1)	8 (8.1)	8 (8.1)	47 (47.5)

Table 3 presents the correlation matrix between fatigue scores (DEFS and DUFS), self-care, and sociodemographic and clinical variables. A significant negative correlation of weak magnitude was observed between age and fatigue on exertion (rho = -0.279; p < 0.01) and age and substantial fatigue (rho = -0.274; p < 0.01). There was a significant positive correlation of moderate magnitude between the NYHA variables and exertional fatigue (rho = 0.501; p < 0.001) and of weak magnitude between NYHA and substantial fatigue (rho = 0.251; p < 0.05). Treatment time had a weak positive correlation with age (rho = 0.272; p < 0.01), and finally, the number of medications showed a moderate positive correlation with the number of comorbidities (rho = 0.337; p < 0.01). In this study, no significant correlation was found between fatigue (DEFES and DUFS) and self-care (p > 0.05).

**Table 3:** Correlation matrix between fatigue, self-care, sociodemographic and clinical variables – João Pessoa, PB, Brazil, 2024.

Variables	1	2	3	4	5	6	7	8	9
1. Self-care	–	–	–	–	–	–	–	–	–
2. DEFS	0.065	–	–	–	–	–	–	–	–
3. DUFS	0.118	–	–	–	–	–	–	–	–
4. Age	-0.003	-0.279**	-0.274**	–	–	–	–	–	–
5. NYHA	0.028	0.501***	0.251*	0.017	–	–	–	–	–
6. Treatment time	-0.074	-0.028	-0.049	0.272**	0.039	–	–	–	–
7. FEVE	-0.021	-0.001	-0.090	0.158	-0.090	0.151	–	–	–
8. Number of comorbidities	0.058	-0.020	0.046	0.199	0.157	0.123	0.042	–	–
9. Number of medications	-0.021	0.167	0.102	0.159	0.129	0.121	-0.122	0.337**	–

Abbreviations: DUFS – *Dutch Fatigue Scale*; DEFS – *Dutch Exertion Fatigue Scale*; NYHA – *New York Heart Association*; FEVE – *Left ventricular ejection fraction*.

*p < 0.05,

**p < 0.01,

***p < 0.001.

## DISCUSSION

Regarding clinical variables, the findings are consistent with the results of a multicenter study conducted in France, Spain, the United Kingdom, Germany, and Italy, which identified a prevalence similar to the clinical profile observed in this research^(12)^.

The findings of this study revealed a high prevalence of substantial fatigue on exertion among the participants. The results corroborate the literature, which indicates that approximately 60–70% of individuals with HF report moderate to high levels of fatigue, highlighting not only the frequency of this symptom but also its persistence throughout the course of the disease^(5)^. Furthermore, research indicates that increased fatigue is associated with a worsening of functional class, suggesting a direct impact on functional capacity and exercise tolerance^(13)^.

In a study conducted in São Paulo, the average total scores for the DUFS and DEFS were higher than those observed in the present study^(13)^. This difference may be related to the severity of HF or the clinical characteristics of the sample, suggesting that patients may be in less advanced stages of the disease or have more effective symptom control.

In the context of heart failure, the combination of systolic and diastolic dysfunction compromises the ability to increase cardiac output. As a consequence, a state of persistent fatigue sets in, in which exhausted patients tend to postpone or omit self-care practices, which worsens symptoms, reinforces apathy, and increases the perception of inability to manage their own treatment. This condition directly impacts the difficulty in adhering to essential activities, such as monitoring body weight, preparing balanced meals, and using medication correctly^(14)^.

Such evidence indicates that fatigue is not only a consequence of the disease, but also a factor that compromises self-care, establishing a vicious cycle with a negative impact on prognosis. Educational interventions on healthy eating, regular moderate physical activity, medication adherence, and energy conservation, including daily activity planning, alternating between activity and rest, relaxation techniques, mobilizing support networks, and adapting the environment, have been employed by nurses in the management of fatigue, contributing to longitudinal follow-up, prevention of complications, and favorable clinical outcomes in patients with heart failure^(15,16,17)^. Therefore, the implementation of culturally appropriate and individualized nursing interventions becomes opportune as an essential component of the therapeutic management of fatigue.

The results demonstrated that the participants in this study presented higher self-care scores than those observed in a national study^(18)^ and in a Spanish study^(19)^, indicating less favorable performance in self-care practices compared to the findings of these studies. These differences may be associated with sociocultural factors, which influence how individuals perceive, understand, and practice self-care, potentially impacting the management of health conditions and, consequently, quality of life^(4)^.

Regarding the frequency of self-care behaviors, it was found that daily weighing, communication about unexpected weight gain, and the practice of regular physical exercise showed the greatest disagreement among participants. These results are similar to research conducted in Switzerland^(20)^. Furthermore, it was found that patients had poor control over their daily fluid intake, consistent with a study conducted in Japan^(21)^. These results affect the maintenance of clinical stability and disease management, which may delay the early recognition of clinical decompensation for decision-making^(22)^.

Conversely, greater agreement was identified in positive self-care behaviors regarding monitoring and reporting symptoms such as shortness of breath, fatigue, and edema to a healthcare professional, resting during the day, restricting salt intake in the diet, regular use of medications, and up-to-date flu vaccination. These findings are similar to those of a study in Slovenia, whose results highlighted that 94% of participants showed good medication adherence and 86% restricted their intake of excess sodium, helping patients to cope positively with HF and keep the disease under control^(23)^.

This study revealed significant correlations between sociodemographic and clinical variables and fatigue scores in HF patients. A negative correlation was observed between age and exertional fatigue, indicating that younger participants reported a greater sense of fatigue during daily activities. One possible explanation is that younger adults tend to experience a greater functional and psychological impact from the limitations imposed by the disease, which may intensify their perception of fatigue. There was a negative correlation between age and substantial fatigue, indicating that younger individuals experienced a higher intensity of generalized fatigue. This finding may be related to individual perceptions of the recognition of HF symptoms. In contrast, older patients may have greater difficulty recognizing these symptoms, since changes resulting from the aging process itself reduce functional reserve and may contribute to increased fatigue, especially in the presence of concomitant chronic diseases such as HF^(24)^.

Furthermore, a positive correlation was found between functional class (NYHA) and exertional fatigue, indicating that the worse the functional classification, the greater the limitation during activities. Similar to this finding, research conducted in Sweden demonstrated, through the use of the instruments RAND 36-item *Item Health Survey* 1.0 (RAND-36v1) and *Work Productivity and Activity Impairment questionnaire* (WPAI), that the worse the NYHA classification is in the HF patient^(25)^, the higher the level of fatigue.

It was found that the older the patient, the longer the treatment time, indicating that older patients tend to be monitored for longer periods. A positive correlation was also observed between the number of comorbidities and the quantity of medications used, showing that the greater the number of associated clinical conditions, the greater the volume of prescribed drugs tends to be. Similar results were found in Japanese research, which associated polypharmacy with a greater number of comorbidities, advanced age, and a worse prognosis in HF patients^(26)^.

These results reinforce the need for nurses to plan care that promotes the appropriate therapeutic use of medications, including identifying weaknesses, preventing the indiscriminate use of drugs, strategies to promote adherence to treatment, family participation in the therapeutic process, and ensuring a safe and effective transition of care.

This study did not find significant correlations between fatigue and self-care. However, the literature records different findings, demonstrating a negative association between these parameters when assessed by instruments such as the *Self-Care of Heart Failure Index version* (SCHFI), suggesting that people with greater fatigue tend to feel less confident in performing self-care behaviors, which negatively impacts their perception of health and disease control^(27)^.

Recent results from a Japanese study show that, although fatigue does not have a direct effect on self-care, this relationship is mediated by perceived control. Therefore, interventions focused on improving the perception of control can positively influence the management of fatigue symptoms^(28)^. Furthermore, the literature indicates that a better self-regulation routine helps in recognizing and managing physical symptoms, but that, depending on the level of fatigue, self-care behavior may be compromised^(29)^.

This study has some limitations; the cross-sectional design does not allow for establishing cause-and-effect relationships. Collecting data at a single center limits the results, and measuring variables through self-reporting is susceptible to recall bias and social desirability bias. Furthermore, the exclusion of patients in NYHA class IV and the magnitude of the observed correlations should be evaluated with caution when generalizing the findings to clinical practice. In this regard, it is recommended that longitudinal studies be developed, conducted in different contexts within the Brazilian scenario and with larger samples, to deepen the understanding of the impact of fatigue on self-care in this population.

Within the context of multidisciplinary clinical practice, this study provides evidence that can support the development of longitudinal interventions in the care of HF patients, especially those aimed at the systematic monitoring of symptoms and the strengthening of self-care. The central role of nursing in this process is highlighted, considering its continuous, close, and educational involvement, which favors the implementation of strategies for energy conservation, fatigue management, promotion of therapeutic adherence, and empowerment of the patient and family for self-care. Adopting these actions is critical to improving clinical monitoring, promoting the stability of health conditions and, consequently, improving clinical outcomes and quality of life.

## CONCLUSION

In this study, fatigue was not associated with self-care behaviors. However, a significant association was observed between age and exertional fatigue, substantial fatigue, and treatment duration. NYHA functional class was also associated with exertional and substantial fatigue. Additionally, a positive association was found between the number of comorbidities and polypharmacy in patients with heart failure, which may directly impact self-care behavior. These findings reinforce the need to implement care plans focused on symptom control to slow disease progression, optimize self-care management, and promote a better quality of life.

## Data Availability

All the data supporting the results of this study were published in the article itself.
