# Supplementary material for: Association between fatigue and self-care in patients with heart failure: a cross-sectional study
Source: Rev Esc Enferm USP. 2026 Sep 25;60:e20250493. doi: 10.1590/1980-220X-REEUSP-2025-0493en (PMC13614708; doi:10.1590/1980-220X-REEUSP-2025-0493en)
Supplement: Translation

# Translation: Associação entre fadiga e autocuidado em pacientes com insuficiência cardíaca: estudo transversal

**DOI:** 10.1590/1980-220X-REEUSP-2025-0493pt

**Authors:** Weslley Italo Ferreira de Oliveira Silva, Yzis Oliveira Pontes Pereira, Filipe Matheus Correia Vieira, Fernanda Kelly Oliveira de Albuquerque, Ricardo Costa da Silva, José Claudio Garcia Lira, Mailson Marques de Sousa

**Affiliations:** 1 Universidade Federal da Paraíba, Departamento de Enfermagem Clínica, Programa de Pós-Graduação em Enfermagem, João Pessoa, PB, Brasil.; 2 Universidade Estadual de Goiás, Departamento de Enfermagem, Ceres, GO, Brasil.; 3 Universidade Federal do Piauí, Departamento de Enfermagem, Programa de Pós-Graduação em Saúde e Comunidade, Floriano, PI, Brasil.

**Keywords:** Autocuidado, Fadiga, Insuficiência Cardíaca, Estudos Transversais

## Abstract

**Objetivo:**

Analisar a associação entre fadiga, autocuidado e variáveis sociodemográficas e clínicas em pacientes com insuficiência cardíaca.

**Método:**

Estudo transversal e correlacional, realizado em um hospital universitário na região Nordeste do Brasil. Foram utilizadas as versões brasileiras da *Dutch Fatigue Scale*, *Dutch Exertion Fatigue Scale* e a *European Heart Failure Self-care Behavior Scale*. A análise incluiu estatística descritiva e correlação de *Spearman*.

**Resultados:**

Participaram 99 pacientes com insuficiência cardíaca. Observou-se fadiga substancial em 71,7% e fadiga ao esforço em 66,7% da amostra. A média da pontuação de autocuidado foi 28,41 ± 7,98. Não houve associação significativa entre fadiga e autocuidado. Identificou-se correlação negativa entre idade e fadiga ao esforço e fadiga substancial; e positiva entre tempo de tratamento e idade. Houve correlação positiva entre a capacidade funcional e fadiga ao esforço e fadiga substancial.

**Conclusão:**

Os achados reforçam a importância da percepção e gerenciamento dos sintomas no autocuidado. Intervenções em saúde são oportunas para a promoção da qualidade de vida desta população.

## INTRODUÇÃO

A insuficiência cardíaca (IC) é uma síndrome clínica caracterizada por alterações estruturais e/ou funcionais do coração, que comprometem o enchimento e/ou a ejeção ventricular, resultando em um débito cardíaco inadequado ou reduzido. Estima-se que 64 milhões de pessoas no cenário global apresentem IC^(1)^. No Brasil, a IC é a principal causa de readmissão hospitalar e de taxas elevadas de mortalidade intra hospitalar, acarretando altos custos financeiros e sobrecarga para o Sistema Único de Saúde^(2)^.

Em pacientes com IC, um dos principais fatores associados à descompensação clínica e à reinternação hospitalar é a baixa adesão ao comportamento de autocuidado^(3)^. O autocuidado é definido como um processo contínuo que envolve a manutenção da saúde a partir da adoção de práticas promotoras de bem-estar e manejo da doença crônica por meio das dimensões de manutenção, monitoramento e gerenciamento^(4)^.

Uma recente revisão sistemática demonstrou que indivíduos com melhores comportamentos de autocuidado apresentam melhor qualidade de vida, além de menores taxas de hospitalização e mortalidade^(5)^. Porém, a ocorrência de sintomas ao longo da experiência de vida pode influenciar o desempenho do autocuidado de maneiras distintas, atuando ora como estímulo para a aquisição de habilidades, ora como inibidor do desejo de engajamento^(4)^.

Pessoas com IC frequentemente apresentam sintomas persistentes, entre os quais a fadiga se destaca por seu caráter subjetivo e debilitante, resultando em comprometimento significativo do funcionamento diário e, especialmente, das condutas de autocuidado. Embora conceituada de modo heterogêneo na literatura^(6)^, a fadiga pode ser categorizada em duas formas distintas que podem ocorrer de maneira simultânea. A fadiga geral, não relacionada ao esforço, e a fadiga por esforço, manifestada durante ou após atividades físicas, caracterizando a intolerância ao exercício^(5)^.

Uma investigação europeia evidenciou que a fadiga de esforço foi significativamente associada à baixa adesão ao autocuidado ao longo do tempo, independente de distúrbios de sono, alterações de humor e outras condições clínicas^(7)^. De forma convergente, uma revisão de escopo que analisou 19 estudos identificou a fadiga como um importante obstáculo às práticas de autocuidado^(6)^. Contudo, apenas um desses estudos foi realizado no Brasil, evidenciando a necessidade de ampliar as pesquisas nacionais sobre o tema.

A fadiga compromete o comportamento de autocuidado ao promover alterações físicas e cognitivas que prejudicam a tomada de decisão em saúde. Indivíduos com IC podem apresentar redução da energia, da concentração e da memória, dificultando o planejamento de atividades diárias relacionadas ao manejo farmacológico e não farmacológico, incluindo o uso regular de medicamentos, o preparo da dieta, a prática de atividade física, o reconhecimento precoce de sinais e sintomas de descompensação clínica e sociais^(5,7)^.

No contexto latino-americano, ainda não há evidências consolidadas que explorem a associação entre a fadiga e o autocuidado em pacientes com IC, o que limita a compressão do fenômeno. Portanto, investigar essa interface é essencial para identificar lacunas de conhecimento, uma vez que os achados poderão subsidiar os profissionais de saúde, especialmente os enfermeiros, no direcionamento de intervenções para o gerenciamento de sintomas e adesão terapêutica. Diante desse contexto, objetivou-se analisar a associação entre fadiga, autocuidado e variáveis sociodemográficas e clínicas em pacientes com insuficiência cardíaca.

## MÉTODO

### Desenho do Estudo

Estudo transversal, desenvolvido no ambulatório de cardiologia de um hospital escola público vinculado ao Sistema Único de Saúde (SUS), localizado na cidade de João Pessoa, Paraíba, Brasil.

### População, Critérios de Seleção e Definição da Amostra

A população do estudo foi constituída por pacientes com IC que estavam em acompanhamento ambulatorial na referida instituição hospitalar. A estimativa amostral foi realizada por meio do software OpenEpi, versão 3.01, tendo-se como base a margem de erro de 5% (erro = 0,05), com grau de confiabilidade de 95%, considerando a proporção verdadeira como de 50% (p = 0,50) em virtude da variabilidade de prevalência de fadiga na literatura^(5)^. Logo, a amostra mínima foi estimada em 94 participantes.

Foram incluídos indivíduos com diagnóstico clínico de IC e idade igual ou superior a 18 anos. Foram excluídos os pacientes classificados na classe IV da New York Heart Association (NYHA), aqueles com diagnóstico de câncer, doenças pulmonares, infarto agudo do miocárdio nos últimos três meses, em tratamento para ansiedade ou depressão, em uso de medicamentos indutores do sono registrados em prontuário, bem como aqueles com déficit cognitivo que comprometesse a compreensão dos objetivos do estudo, a resposta ou o preenchimento dos instrumentos de coleta. Pacientes classificados na classe IV foram excluídos em razão da sintomatologia avançada da IC^(2)^. Também foram excluídos os pacientes desorientados quanto ao tempo, espaço ou pessoa, condição avaliada pela observação dos pesquisadores e por meio das perguntas: ‘Qual o seu nome completo?’, ‘Qual é a sua idade?’, ‘Que dia é hoje?’ e ‘Onde estamos neste momento?’.

### Coleta de Dados

Os dados foram coletados por dois graduandos de enfermagem devidamente treinados, no período de março a novembro de 2024 por meio de entrevista individual, em ambiente reservado e com aplicação de três instrumentos, a saber:

Para caracterização da amostra foi utilizado um formulário^(2)^ contendo variáveis sociodemográficas e clínicas: procedência, idade (em anos), sexo, cor da pele autodeclarada, escolaridade, estado civil, renda familiar, situação ocupacional, etiologia da IC, tempo desde o diagnóstico, classe funcional da IC segundo a NYHA, fração de ejeção do ventrículo esquerdo (FEVE) registrada em laudo de ecocardiograma transtorácico realizado nos últimos seis meses, comorbidades crônicas associadas, terapêutica medicamentosa em uso e histórico de hospitalizações de emergência.

Para avaliação da fadiga foram utilizadas as versões traduzidas e adaptadas para o português das escalas *Dutch Fatigue Scale* (DUFS) e *Dutch Exertion Fatigue Scale* (DEFS)^(8)^. A DUFS é composta por oito itens que mensuram a frequência da fadiga nos últimos três a seis meses anteriores à coleta de dados. Essa escala avalia aspectos como fadiga geral, redução da concentração, diminuição da motivação ou interesse, dificuldade para iniciar ou manter atividades diárias e a percepção do indivíduo acerca da intensidade da fadiga física e mental. As respostas seguem formato Likert de cinco pontos, variando de 1 (nunca) a 5 (sempre). A pontuação total varia de 8 a 40, valores mais altos indicam maior intensidade de fadiga^(8)^.

A DEFS é composta por nove itens que avaliam a frequência da fadiga durante a realização de atividades cotidianas, como caminhar, realizar compras ou recolher o lixo. As respostas seguem uma escala do tipo Likert de cinco pontos, variando de 1 (atividade não provoca fadiga) a 5 (atividade extremamente fatigante). A pontuação total pode variar entre 9 e 45 pontos, com maiores valores indicando maior intensidade da fadiga. As escalas mostraram alfa de Cronbach com valores de 0,84 para DUFS e 0,92 para DEFS^(8)^.

Pesquisadores brasileiros sugerem pontos de corte para interpretação das escalas: ≥ 14,5 na DUFS para caracterizar ‘fadiga substancial’ e ≥ 12,5 na DEFS para ‘fadiga substancial ao esforço’. Ressalta-se que valores inferiores não devem ser interpretados como ausência de fadiga, mas como níveis menos expressivos. Esses parâmetros foram previamente utilizados em estudo nacional^(9)^.

O comportamento de autocuidado foi avaliado pela *European Heart Failure Self-care Behavior Scale* (EHFScBS), traduzida e validada para o português do Brasil^(10)^. O instrumento contém 12 itens em um único domínio, com respostas em escala Likert de 1 (“concordo plenamente”) a 5 (“discordo plenamente”), totalizando escores entre 12 e 60 pontos, em que valores mais elevados indicam pior comportamento de autocuidado. A estimativa de confiabilidade apresentou alfa de Cronbach de 0,70 e coeficiente de correlação intraclasse de 0,87^(10)^.

### Análise e Tratamento dos Dados

Os dados foram organizados em planilha Excel e analisados no software Jamovi, versão 2.6. Empregou-se estatística descritiva (frequências absolutas e relativas para variáveis categóricas; média, desvio-padrão, mediana e intervalo interquartil para variáveis numéricas) e inferencial. A normalidade foi verificada pelo teste de Shapiro-Wilk, que indicou distribuição não paramétrica. Para verificar as relações entre as variáveis sociodemográficas, clínicas, fadiga e autocuidado, aplicou se o coeficiente de correlação de Spearman. A força das correlações foi classificada como fraca (0,1–0,3), moderada (0,4–0,6) ou forte (0,7–0,9)^(11)^. Adotou-se nível de significância de 5% e um valor de p < 0,05.

### Aspectos Éticos

O estudo foi aprovado pelo Comitê de Ética em Pesquisa (CEP) do Hospital Universitário Lauro Wanderley sob o parecer n.º 6.704.645. Todos os participantes receberam informações verbais e escritas sobre a pesquisa e confirmaram sua concordância por meio da assinatura do Termo de Consentimento Livre e Esclarecido (TCLE), em duas vias.

## RESULTADOS

Participaram do estudo 99 pacientes com IC, sendo a maioria do sexo masculino (n = 52; 52,5%), pardos (n = 59; 59,6%), casados (n = 54; 54,5%), aposentados (n = 55; 55,6%), com renda de até um salário mínimo brasileiro (n = 68; 68,7%), e procedentes de João Pessoa, PB (n = 66; 66,7%). A média de idade dos participantes foi de 59,33 (DP = 10,47) e 7,04 (DP = 5,21) anos de estudo.

Quanto às variáveis clínicas, 74 (74,7%) possuíam IC de etiologia isquêmica, 46 (46,5%) foram categorizados na classe II da NYHA, com FEVE média de 37,57% (DP = 12,86). A hipertensão arterial foi a comorbidade mais prevalente associada à IC (n = 67;67,7%). Cerca de 62 (62,6%) praticavam atividade física e 89 (89,9%) informaram que não ingeriam bebidas alcoólicas.

Utilizando a DUFS, cerca de 71,7% dos pacientes apresentaram fadiga substancial (≥ 14,5), enquanto na DEFS, 66,7% apresentaram fadiga substancial ao esforço (≥ 12,5). Por sua vez, o autocuidado foi de 28,41 (DP = 7,98), segundo a EHFScBS, conforme a Tabela 1.

**Tabela 1:** Escores dos instrumentos DUFS, DEFS e EHFScBS – João Pessoa, PB, Brasil, 2024.

Variáveis	Média ± desviopadrão	Valor mínimo	Valor máximo	Mediana
Escore DUFS	20,92 ± 8,11	8	40	21,00
Escore DEFS	17,99 ± 8,35	9	45	16,00
Escore EHFScBS	28,41 ± 7,98	14	48	27,00

Abreviaturas: DUFS – *Dutch Fatigue Scale*; DEFS – *Dutch Exertion Fatigue Scale*; EHFScBS – *European Heart Failure Self-care Behavior Scale*.

Na Tabela 2, os itens “Eu me peso todos os dias” (item 1), “Se eu ganhar 2kg em 1 semana, entro em contato com meu médico ou com a enfermeira (ou algum serviço de saúde)” (item 5), “Limito a quantidade de líquidos que bebo (não mais do que 1,5 a 2L por dia)” (item 6) e “Faço exercícios regularmente” (item 12) apresentaram maior pontuação, demonstrando uma baixa adesão. Já os itens 2, 3, 4, 7, 8, 9, 10 e 11 indicaram menor pontuação, o que representa uma melhor concordância para as atividades de autocuidado.

**Tabela 2:** Distribuição da frequência de respostas da escala de autocuidado (EHFScBS) – João Pessoa, PB, Brasil, 2024.

Questões	Concordo plenamente n (%)	Quase sempre concordo n (%)	Às vezes concordo n (%)	Quase nunca concordo n (%)	Discordo plenamente n (%)
1. Eu me peso todos os dias.	8 (8,1)	10 (10,1)	18 (18,2)	12 (12,1)	51 (51,5)
2. Se sinto falta de ar, repouso.	68 (68,7)	2 (2,0)	5 (5,1)	7 (7,1)	17 (17,2)
3. Se minha falta de ar aumenta, entro em contato com meu médico ou com a enfermeira (ou algum serviço de saúde).	62 (62,6)	7 (7,1)	4 (4,0)	10 (10,1)	16 (16,2)
4. Se meus pés ou pernas tornarem-se mais inchados mais do que de costume, entro em contato com meu médico ou com a enfermeira (ou algum serviço de saúde).	69 (69,7)	3 (3,0)	1 (1,0)	3 (3,0)	23 (23,2)
5. Se eu ganhar 2kg em 1 semana, entro em contato com meu médico ou com a enfermeira (ou algum serviço de saúde).	40 (40,4)	7 (7,1)	3 (3,0)	7 (7,1)	42 (42,4)
6. Limito a quantidade de líquidos que bebo (não mais do que 1,5 a 2L por dia).	29 (29,3)	7 (7,1)	7 (7,1)	3 (3,0)	53 (53,5)
7. Descanso durante o dia.	79 (79,8)	5 (5,1)	8 (8,1)	1 (1,0)	6 (6,1)
8. Se meu cansaço aumenta, entro em contato com meu médico ou com a enfermeira (ou algum serviço de saúde).	75 (75,8)	7 (7,1)	5 (5,1)	2 (2,0)	10 (10,1)
9. Ingiro uma dieta com pouco sal.	76 (76,8)	5 (5,1)	7 (7,1)	3 (3,0)	8 (8,1)
10. Tomo meu medicamento de acordo com a prescrição.	83 (83,8)	6 (6,1)	5 (5,1)	2 (2,0)	3 (3,0)
11. Tomo a vacina contra a gripe todos os anos.	57 (57,6)	7 (7,1)	3 (3,0)	5 (5,1)	27 (27,3)
12. Faço exercícios regularmente.	26 (26,3)	10 (10,1)	8 (8,1)	8 (8,1)	47 (47,5)

A Tabela 3 apresenta a matriz de correlação entre os escores de fadiga (DEFS e DUFS), autocuidado e variáveis sociodemográficas e clínicas. Observou-se correlação negativa significativa de fraca magnitude entre idade e fadiga ao esforço (rho = −0,279; p < 0,01) e com fadiga substancial (rho = −0,274; p < 0,01). Houve correlação significativa positiva de moderada magnitude entre as variáveis NYHA e fadiga ao esforço (rho = 0,501; p < 0,001) e de fraca magnitude entre NYHA e fadiga substancial (rho = 0,251; p < 0,05). O tempo de tratamento teve correlação fraca positiva com a idade (rho = 0,272; p < 0,01) e, por fim, o número de medicamentos teve correlação positiva moderada quanto ao número de comorbidades (rho = 0,337; p < 0,01). Neste estudo, não foi encontrada correlação significativa entre fadiga (DEFES e DUFS) e autocuidado (p > 0,05).

**Tabela 3:** Matriz de correlações entre fadiga, autocuidado, variáveis sociodemográficas e clínicas – João Pessoa, PB, Brasil, 2024.

Variáveis	1	2	3	4	5	6	7	8	9
1. Autocuidado	–	–	–	–	–	–	–	–	–
2. DEFS	0,065	–	–	–	–	–	–	–	–
3. DUFS	0,118	–	–	–	–	–	–	–	–
4. Idade	-0,003	-0,279**	-0,274**	–	–	–	–	–	–
5. NYHA	0,028	0,501***	0,251*	0,017	–	–	–	–	–
6. Tempo de tratamento	-0,074	-0,028	-0,049	0,272**	0,039	–	–	–	–
7. FEVE	-0,021	-0,001	-0,090	0,158	-0,090	0,151	–	–	–
8. Número de comorbidades	0,058	-0,020	0,046	0,199	0,157	0,123	0,042	–	–
9. Número de medicamentos	-0,021	0,167	0,102	0,159	0,129	0,121	-0,122	0,337**	–

Abreviaturas: DUFS – *Dutch Fatigue Scale*; DEFS – *Dutch Exertion Fatigue Scale*; NYHA – *New York Heart Association*; FEVE – *Fração de ejeção do ventrículo esquerdo*.

*p < 0,05,

**p < 0,01,

***p < 0,001.

## DISCUSSÃO

No que se refere às variáveis clínicas, os achados são consistentes com os resultados de um estudo multicêntrico realizado na França, Espanha, Reino Unido, Alemanha e Itália, que identificou prevalência semelhante ao perfil clínico observado nesta pesquisa^(12)^.

Os achados deste estudo evidenciaram uma elevada prevalência de fadiga substancial ao esforço entre os participantes. Os resultados corroboram com a literatura, que aponta que cerca de 60‒70% dos indivíduos com IC apresentam relatos de níveis moderados a elevados de fadiga, o que evidencia não apenas a frequência desse sintoma, mas também sua persistência ao longo do curso da doença^(5)^. Além disso, investigação aponta que o aumento da fadiga está associado à piora da classe funcional, sugerindo impacto direto na capacidade funcional e na tolerância ao esforço^(13)^.

Em uma pesquisa realizada em São Paulo, os escores totais médios da DUFS e da DEFS foram superiores aos observados no presente estudo^(13)^. Essa diferença pode estar relacionada à gravidade da IC ou às características clínicas da amostra, sugerindo que os pacientes possam estar em estágios menos avançados da doença ou com controle mais efetivo dos sintomas.

No contexto da IC, a combinação de disfunção sistólica e diastólica compromete a capacidade de aumento do débito cardíaco. Como consequência, instala-se um quadro de fadiga persistente, no qual pacientes exaustos tendem a adiar ou omitir práticas de autocuidado, o que agrava os sintomas, reforça a apatia e aumenta a percepção de incapacidade de gerir o próprio tratamento. Essa condição repercute diretamente na dificuldade de adesão a atividades essenciais, como o monitoramento do peso corporal, a preparação de refeições balanceadas e o uso correto da medicação^(14)^.

Tais evidências indicam que a fadiga não é apenas uma consequência da doença, mas também um fator que compromete o autocuidado, instaurando um ciclo vicioso com impacto negativo sobre o prognóstico. Intervenções educativas sobre alimentação saudável, prática regular de atividades físicas moderadas, adesão medicamentosa e conservação de energia incluindo planejamento das atividades diárias, alternância entre atividade e repouso, técnicas de relaxamento, mobilização de rede de apoio e adequação do ambiente têm sido empregadas por enfermeiros no manejo da fadiga, contribuindo para o acompanhamento longitudinal, a prevenção de complicações e desfechos clínicos favoráveis em pacientes com IC^(15,16,17)^. Assim, torna-se oportuna a implantação de intervenções de enfermagem culturalmente adequadas e individualizadas como componente essencial do manejo terapêutico da fadiga.

Os resultados demonstraram que os participantes deste estudo apresentaram escores de autocuidado superiores aos observados em um estudo nacional^(18)^ e em um estudo espanhol^(19)^, o que indica um desempenho menos favorável nas práticas de autocuidado em comparação aos achados destas pesquisas. Tais diferenças podem estar associadas a fatores socioculturais, que influenciam a forma como os indivíduos percebem, compreendem e praticam o autocuidado, podendo repercutir no controle da condição de saúde e, consequentemente, na qualidade de vida^(4)^.

No que se refere à frequência de comportamentos de autocuidado, verificou-se que a pesagem diária, a comunicação sobre o ganho de peso inesperado e a prática de exercícios físicos regulares apresentaram maior discordância entre os participantes. Esses resultados assemelham-se a pesquisa realizada na Suíça^(20)^. Além disso, constatou-se que os pacientes apresentavam baixo controle da ingesta diária de líquidos, em consonância com um estudo conduzido no Japão^(21)^. Tais resultados afetam a manutenção da estabilidade clínica e gerenciamento da doença, o que pode retardar o reconhecimento precoce de descompensação clínica para tomada de decisão^(22)^.

Por outro lado, identificou-se maior concordância em comportamentos de autocuidado positivos com relação ao monitoramento e relato para um profissional de saúde de sintomas como falta de ar, cansaço e edema, descanso durante o dia, restrição do consumo de sal na dieta, uso regular das medicações e vacina contra a gripe atualizada, que se assemelham às variáveis encontradas em pesquisa na Eslovênia, cujos resultados destacaram que 94% dos participantes apresentaram boa adesão medicamentosa e 86% restringiam o consumo de sódio em excesso, auxiliando o paciente a lidar positivamente com a IC e manter a doença de forma controlada^(23)^.

Este estudo evidenciou correlações significativas entre variáveis sociodemográficas, clínicas e os escores de fadiga em pacientes com IC. Observou-se correlação negativa entre idade e fadiga ao esforço, indicando que participantes mais jovens relataram maior sensação de cansaço durante atividades cotidianas. Uma possível explicação é que adultos mais jovens tendem a vivenciar maior impacto funcional e psicológico diante das limitações impostas pela doença, o que pode intensificar a percepção de fadiga. Houve correlação negativa entre a idade e a fadiga substancial, indicando que indivíduos mais jovens apresentaram maior intensidade de fadiga generalizada. Esse achado pode estar relacionado à percepção individual acerca do reconhecimento dos sintomas da IC. Em contrapartida, pacientes idosos podem apresentar maior dificuldade em reconhecer esses sintomas, uma vez que as alterações decorrentes do próprio processo de envelhecimento reduzem a reserva funcional e podem contribuir para o aumento da fadiga, sobretudo na presença concomitante de doenças crônicas na IC^(24)^.

Além disso, verificou-se correlação positiva entre a classe funcional (NYHA) e a fadiga ao esforço, indicando que quanto pior a classificação funcional, maior a limitação durante as atividades. Semelhante a esse achado, uma pesquisa realizada na Suécia demonstrou, por meio dos instrumentos RAND 36-*Item Health Survey* 1.0 (RAND-36v1) e *Work Productivity and Activity Impairment questionnaire* (WPAI), que o nível de fadiga tende a aumentar quanto pior for a classificação NYHA no paciente com IC^(25)^.

Identificou-se que quanto maior a idade do paciente, maior é o tempo de tratamento, indicando que pacientes mais idosos tendem a estar em acompanhamento por períodos mais prolongados. Observou-se, também, correlação positiva entre o número de comorbidades e a quantidade de medicamentos utilizados, evidenciando que quanto maior o número de condições clínicas associadas, maior tende a ser o volume de fármacos prescritos. Resultados semelhantes foram encontrados em pesquisa japonesa, que associou a polifarmácia a um maior número de comorbidades, idade avançada e pior prognóstico em pacientes com IC^(26)^.

Esses resultados reforçam a necessidade de que o enfermeiro planeje uma assistência que favoreça o uso terapêutico adequado dos medicamentos, incluindo a identificação de fragilidades, prevenção do uso indiscriminado de fármacos, estratégias para promover a adesão ao tratamento, participação da família no processo terapêutico e garantia de uma transição de cuidados segura e efetiva.

Nesta pesquisa não foram obtidas correlações significativas entre fadiga e autocuidado. No entanto, a literatura registra achados distintos, demonstrando associação negativa entre esses parâmetros quando avaliados por instrumentos como o *Self-Care of Heart Failure Index version* (SCHFI), sugerindo que pessoas com maior fadiga tendem a se sentir menos confiantes para desempenhar comportamentos de autocuidado, o que repercute negativamente na percepção de saúde e no controle da doença^(27)^.

Resultados recentes de um estudo japonês mostram que, embora a fadiga não tenha efeito direto sobre o autocuidado, essa relação é mediada pelo controle percebido. Logo, intervenções com foco na melhoria da percepção de controle podem influenciar positivamente o gerenciamento dos sintomas da fadiga^(28)^. Ainda, a literatura aponta que uma melhor rotina de autorregulação auxilia no reconhecimento e na administração de sintomas físicos, mas que, a depender do nível de fadiga, o comportamento de autocuidado pode ser comprometido^(29)^.

Este estudo apresenta algumas limitações, o delineamento transversal não permite estabelecer relações de causa e efeito. A coleta de dados em um único centro restringe os resultados, e a mensuração das variáveis por meio de autorrelato está suscetível a vieses de memória e desejabilidade social. Ademais, a exclusão de pacientes em classe IV da NYHA e a magnitude das correlações observadas devem ser avaliadas com cautela na generalização dos achados para a prática clínica. Nesse sentido, recomenda-se o desenvolvimento de estudos longitudinais, conduzidos em diferentes contextos do cenário brasileiro e com amostras mais amplas, para aprofundar a compreensão sobre o impacto da fadiga no autocuidado desta população.

No âmbito da prática clínica multiprofissional, este estudo oferece evidências que podem subsidiar o desenvolvimento de intervenções longitudinais no cuidado a pessoas com IC, especialmente aquelas direcionadas ao monitoramento sistemático dos sintomas e ao fortalecimento do autocuidado. Destaca-se o papel central da enfermagem nesse processo, considerando sua atuação contínua, próxima e educativa, que favorece a implementação de estratégias para conservação de energia, manejo da fadiga, promoção da adesão terapêutica e capacitação do paciente e família para o autocuidado. A adoção dessas ações é fundamental para qualificar o acompanhamento clínico, favorecer a estabilidade da condição de saúde e, consequentemente, melhorar desfechos clínicos e a qualidade de vida.

## CONCLUSÃO

Neste estudo, a fadiga não apresentou associação com o comportamento de autocuidado. Entretanto, observou-se associação significativa da idade com a fadiga ao esforço, a fadiga substancial e o tempo de tratamento. A classe funcional da NYHA também se mostrou associada à fadiga ao esforço e à fadiga substancial. Adicionalmente, verificou-se associação positiva entre o número de comorbidades e a polifarmácia em pacientes com insuficiência cardíaca, aspecto que pode impactar diretamente o comportamento de autocuidado. Esses achados reforçam a necessidade de implementar planos de cuidado direcionados ao controle dos sintomas, com vistas a retardar a progressão da doença, otimizar o gerenciamento do autocuidado e promover melhor qualidade de vida.

## Data Availability

Todo o conjunto de dados que dá suporte aos resultados deste estudo foi publicado no próprio artigo.

# Translation: Asociación entre la fatiga y el autocuidado en pacientes con insuficiencia cardíaca: estudio transversal

**DOI:** 10.1590/1980-220X-REEUSP-2025-0493es

**Authors:** Weslley Italo Ferreira de Oliveira Silva, Yzis Oliveira Pontes Pereira, Filipe Matheus Correia Vieira, Fernanda Kelly Oliveira de Albuquerque, Ricardo Costa da Silva, José Claudio Garcia Lira, Mailson Marques de Sousa

**Affiliations:** 1 Universidade Federal da Paraíba, Departamento de Enfermagem Clínica, Programa de Pós-Graduação em Enfermagem, João Pessoa, PB, Brasil.; 2 Universidade Estadual de Goiás, Departamento de Enfermagem, Ceres, GO, Brasil.; 3 Universidade Federal do Piauí, Departamento de Enfermagem, Programa de Pós-Graduação em Saúde e Comunidade, Floriano, PI, Brasil.

**Keywords:** Autocuidado, Fatiga, Insuficiencia Cardíaca, Estudios Transversales

## Abstract

**Objetivo:**

Analizar la asociación entre la fatiga, el autocuidado y las variables sociodemográficas y clínicas en pacientes con insuficiencia cardíaca.

**Método:**

Estudio transversal y correlacional, realizado en un hospital universitario de la región noreste de Brasil. Se utilizaron las versiones brasileñas de la *Dutch Fatigue Scale*, la *Dutch Exertion Fatigue Scale* y la *European Heart Failure Self-care Behavior Scale*. El análisis incluyó estadísticas descriptivas y la correlación de *Spearman*.

**Resultados:**

Participaron 99 pacientes con insuficiencia cardíaca. Se observó fatiga sustancial en el 71,7 % y fatiga de esfuerzo en el 66,7 % de la muestra. El promedio de la puntuación de autocuidado fue de 28,41 ± 7,98. No se encontró una asociación significativa entre la fatiga y el autocuidado. Se identificó una correlación negativa entre la edad y la fatiga por esfuerzo y la fatiga sustancial; y una correlación positiva entre la duración del tratamiento y la edad. Hubo una correlación positiva entre la capacidad funcional y la fatiga por esfuerzo, y entre la capacidad funcional y la fatiga sustancial.

**Conclusión:**

Los hallazgos refuerzan la importancia de la percepción y el manejo de los síntomas en el autocuidado. Las intervenciones en salud son oportunas para promover la calidad de vida de esta población.

## INTRODUCCIÓN

La insuficiencia cardíaca (IC) es un síndrome clínico caracterizado por alteraciones estructurales y/o funcionales del corazón, que comprometen el llenado y/o la eyección ventricular, lo que da lugar a un gasto cardíaco inadecuado o reducido. Se estima que 64 millones de personas en todo el mundo padecen IC^(1)^. En Brasil, la IC es la principal causa de reingreso hospitalario y de altas tasas de mortalidad intrahospitalaria, lo que genera altos costos financieros y una sobrecarga para el Sistema Único de Salud^(2)^.

En pacientes con IC, uno de los principales factores asociados a la descompensación clínica y a la reingreso hospitalario es la baja adherencia al autocuidado^(3)^. El autocuidado se define como un proceso continuo que implica el mantenimiento de la salud a partir de la adopción de prácticas que promueven el bienestar y el manejo de la enfermedad crónica a través de las dimensiones de mantenimiento, monitoreo y gestión^(4)^.

Una revisión sistemática reciente demostró que las personas con mejores prácticas de autocuidado presentan una mejor calidad de vida, además de menores tasas de hospitalización y mortalidad^(5)^. Sin embargo, la aparición de síntomas a lo largo de la vida puede influir en el desempeño del autocuidado de diversas maneras, actuando a veces como estímulo para la adquisición de habilidades y otras como inhibidor del deseo de participar^(4)^.

Las personas con IC suelen presentar síntomas persistentes, entre los cuales la fatiga se destaca por su carácter subjetivo y debilitante, lo que resulta en un deterioro significativo del funcionamiento diario y, especialmente, de las conductas de autocuidado. Aunque se conceptualiza de manera heterogénea en la literatura^(6)^, la fatiga puede clasificarse en dos formas distintas que pueden ocurrir simultáneamente. La fatiga general, no relacionada con el esfuerzo, y la fatiga por esfuerzo, que se manifiesta durante o después de actividades físicas y caracteriza la intolerancia al ejercicio^(5)^.

Una investigación europea demostró que la fatiga por esfuerzo se asoció significativamente con una baja adherencia al autocuidado a lo largo del tiempo, independientemente de los trastornos del sueño, los cambios de estado de ánimo y otras afecciones clínicas^(7)^. De manera convergente, una revisión de alcance que analizó 19 estudios identificó la fatiga como un obstáculo importante para las prácticas de autocuidado^(6)^. Sin embargo, solo uno de esos estudios se llevó a cabo en Brasil, lo que pone de manifiesto la necesidad de ampliar las investigaciones nacionales sobre el tema.

La fatiga afecta el comportamiento de autocuidado al provocar alteraciones físicas y cognitivas que perjudican la toma de decisiones en materia de salud. Las personas con IC pueden presentar una disminución de la energía, la concentración y la memoria, lo que dificulta la planificación de las actividades diarias relacionadas con el manejo farmacológico y no farmacológico, incluyendo el uso regular de medicamentos, la preparación de la dieta, la práctica de actividad física y el reconocimiento temprano de los signos y síntomas de descompensación clínica y social^(5,7)^.

En el contexto latinoamericano, aún no existen evidencias consolidadas que exploren la asociación entre la fatiga y el autocuidado en pacientes con IC, lo que limita la comprensión del fenómeno. Por lo tanto, investigar esta relación es esencial para identificar brechas de conocimiento, ya que los hallazgos podrían servir de apoyo a los profesionales de la salud, especialmente a los enfermeros, a la hora de orientar las intervenciones para el manejo de los síntomas y la adherencia terapéutica. Ante este contexto, se propuso analizar la asociación entre la fatiga, el autocuidado y las variables sociodemográficas y clínicas en pacientes con insuficiencia cardíaca.

## MÉTODO

### Diseño del Estudio

Estudio transversal, realizado en la clínica ambulatoria de cardiología de un hospital universitario público adscrito al Sistema Único de Salud (SUS), ubicado en la ciudad de João Pessoa, Paraíba, Brasil.

### Población, Criterios de Selección y Definición de la Muestra

La población del estudio estuvo compuesta por pacientes con IC que se encontraban en seguimiento ambulatorio en dicha institución hospitalaria. La estimación de la muestra se realizó mediante el software OpenEpi, versión 3.01, basándose en un margen de error del 5% (error = 0,05), con un nivel de confianza del 95%, considerando la proporción verdadera como del 50% (p = 0,50) debido a la variabilidad de la prevalencia de la fatiga en la literatura^(5)^. Por lo tanto, la muestra mínima se estimó en 94 participantes.

Se incluyeron personas con diagnóstico clínico de insuficiencia cardíaca y edad igual o superior a 18 años. Se excluyeron los pacientes clasificados en la clase IV de la Asociación Cardíaca de Nueva York (NYHA), aquellos con diagnóstico de cáncer, enfermedades pulmonares, infarto agudo de miocardio en los últimos tres meses, en tratamiento para la ansiedad o la depresión, que tomaran medicamentos inductores del sueño registrados en el expediente médico, así como aquellos con déficit cognitivo que comprometiera la comprensión de los objetivos del estudio, la respuesta o el llenado de los instrumentos de Recolección de datos. Los pacientes clasificados en la clase IV fueron excluidos debido a la sintomatología avanzada de la insuficiencia cardíaca^(2)^. También se excluyó a los pacientes desorientados en cuanto al tiempo, el espacio o las personas, condición evaluada mediante la observación de los investigadores y a través de las preguntas: “¿Cuál es su nombre completo?”, “¿Cuántos años tiene?”, “¿Qué día es hoy?” y “¿Dónde estamos en este momento?”.

### Recolección de Datos

Los datos fueron recopilados por dos estudiantes de enfermería debidamente capacitados, durante el período de marzo a noviembre de 2024, mediante entrevistas individuales en un ambiente privado y con la aplicación de tres instrumentos, a saber.

Para la caracterización de la muestra se utilizó un formulario^(2)^ que contenía variables sociodemográficas y clínicas: procedencia, edad (en años), sexo, color de piel autodeclarado, nivel educativo, estado civil, ingreso familiar, situación laboral, etiología de la IC, tiempo transcurrido desde el diagnóstico, clase funcional de la IC según la NYHA, fracción de eyección del ventrículo izquierdo (FEVI) registrada en el informe de un ecocardiograma transtorácico realizado en los últimos seis meses, comorbilidades crónicas asociadas, tratamiento farmacológico en uso e historial de hospitalizaciones de emergencia.

Para evaluar la fatiga se utilizaron las versiones traducidas y adaptadas al portugués de las escalas *Dutch Fatigue Scale* (DUFS) y *Dutch Exertion Fatigue Scale* (DEFS)^(8)^. La DUFS consta de ocho ítems que miden la frecuencia de la fatiga en los últimos tres a seis meses previos a la recolección de datos. Esta escala evalúa aspectos como la fatiga general, la disminución de la concentración, la reducción de la motivación o el interés, la dificultad para iniciar o mantener las actividades cotidianas y la percepción del individuo sobre la intensidad de la fatiga física y mental. Las respuestas siguen un formato de Likert de cinco puntos, que varía de 1 (nunca) a 5 (siempre). La puntuación total varía de 8 a 40; los valores más altos indican una mayor intensidad de fatiga^(8)^.

La DEFS consta de nueve ítems que evalúan la frecuencia de la fatiga al realizar actividades cotidianas, como caminar, ir de compras o sacar la basura. Las respuestas siguen una escala tipo Likert de cinco puntos, que varía de 1 (la actividad no provoca fatiga) a 5 (la actividad es extremadamente fatigante). La puntuación total puede variar entre 9 y 45 puntos; los valores más altos indican una mayor intensidad de la fatiga. Las escalas mostraron un alfa de Cronbach con valores de 0,84 para la DUFS y de 0,92 para la DEFS^(8)^.

Investigadores brasileños sugieren puntos de corte para la interpretación de las escalas: ≥ 14,5 en la DUFS para caracterizar “fatiga sustancial” y ≥ 12,5 en la DEFS para “fatiga sustancial ante el esfuerzo”. Cabe destacar que los valores inferiores no deben interpretarse como ausencia de fatiga, sino como niveles menos significativos. Estos parámetros se utilizaron previamente en un estudio nacional^(9)^.

El comportamiento de autocuidado se evaluó mediante la *European Heart Failure Self-care Behavior Scale* (EHFScBS), traducida y validada al portugués de Brasil^(10)^. El instrumento contiene 12 ítems en un único dominio, con respuestas en una escala de Likert de 1 (“estoy totalmente de acuerdo”) a 5 (“estoy totalmente en desacuerdo”), lo que da un total de puntuaciones entre 12 y 60 puntos, en las que los valores más altos indican un peor comportamiento de autocuidado. La estimación de la confiabilidad presentó un alfa de Cronbach de 0,70 y un coeficiente de correlación intraclase de 0,87^(10)^.

### Análisis y Procesamiento de los Datos

Los datos se organizaron en una hoja de cálculo de Excel y se analizaron con el software Jamovi, versión 2.6. Se emplearon estadísticas descriptivas (frecuencias absolutas y relativas para las variables categóricas; media, desviación estándar, mediana e intervalo intercuartílico para las variables numéricas) e inferenciales. La normalidad se verificó mediante la prueba de Shapiro-Wilk, que indicó una distribución no paramétrica. Para verificar las relaciones entre las variables sociodemográficas, clínicas, la fatiga y el autocuidado, se aplicó el coeficiente de correlación de Spearman. La fuerza de las correlaciones se clasificó como débil (0,1–0,3), moderada (0,4–0,6) o fuerte (0,7–0,9)^(11)^. Se adoptó un nivel de significación del 5% y un valor de p < 0,05.

### Aspectos Éticos

El estudio fue aprobado por el Comité de Ética en Investigación (CEP) del Hospital Universitario Lauro Wanderley mediante el dictamen n.º 6.704.645. Todos los participantes recibieron información verbal y escrita sobre la investigación y confirmaron su consentimiento mediante la firma del Término de Consentimiento Libre e Informado (TCLE), en dos ejemplares.

## RESULTADOS

Participaron en el estudio 99 pacientes con insuficiencia cardíaca (IC), la mayoría de los cuales eran hombres (n = 52; 52,5%), de raza mestiza (n = 59; 59,6%), casados (n = 54; 54,5%), jubilados (n = 55; 55,6%), con ingresos de hasta un salario mínimo brasileño (n = 68; 68,7%) y residentes en João Pessoa, PB (n = 66; 66,7%). La edad promedio de los participantes fue de 59,33 (DE = 10,47) años y el promedio de años de estudio fue de 7,04 (DE = 5,21).

En cuanto a las variables clínicas, 74 (74,7%) presentaban IC de etiología isquémica; 46 (46,5%) fueron clasificados en la clase II de la NYHA, con una FEVI promedio de 37,57% (DE = 12,86). La hipertensión arterial fue la comorbilidad más prevalente asociada a la IC (n = 67; 67,7%). Aproximadamente 62 (62,6%) realizaban actividad física y 89 (89,9%) informaron que no consumían bebidas alcohólicas.

Al utilizar la escala DUFS, alrededor del 71,7% de los pacientes presentaron fatiga sustancial (≥ 14,5), mientras que en la escala DEFS, el 66,7% presentó fatiga sustancial al esfuerzo (≥ 12,5). Por su parte, el autocuidado fue de 28,41 (DE = 7,98), según la EHFScBS, tal como se muestra en la Tabla 1.

**Tabla 1:** Puntuaciones de los instrumentos DUFS, DEFS y EHFScBS – João Pessoa, PB, Brasil, 2024.

Variables	Media ± desviación estándar	Valor mínimo	Valor máximo	Mediana
Escore DUFS	20,92 ± 8,11	8	40	21,00
Escore DEFS	17,99 ± 8,35	9	45	16,00
Escore EHFScBS	28,41 ± 7,98	14	48	27,00

Abreviaturas: DUFS – *Dutch Fatigue Scale*; DEFS – *Dutch Exertion Fatigue Scale*; EHFScBS – *European Heart Failure Self-care Behavior Scale*.

En la Tabla 2, los ítems “Me peso todos los días” (ítem 1), “Si aumento 2 kg en una semana, me comunico con mi médico o con la enfermera (o con algún servicio de salud)” (ítem 5), “Limito la cantidad de líquidos que bebo (no más de 1,5 a 2 L al día)” (ítem 6) y “Hago ejercicio con regularidad” (ítem 12) obtuvieron las puntuaciones más altas, lo que demuestra una baja adherencia. Por su parte, los ítems 2, 3, 4, 7, 8, 9, 10 y 11 obtuvieron puntuaciones más bajas, lo que representa una mejor adherencia a las actividades de autocuidado.

**Tabla 2:** Distribución de la frecuencia de las respuestas de la escala de autocuidado (EHFScBS) – João Pessoa, PB, Brasil, 2024.

Preguntas	Concuerdo plenamente n (%)	Casi siempre concuerdo n (%)	A veces concuerdo n (%)	Casi nunca concuerdo n (%)	Plenamente en desacuerdo n (%)
1. Me peso todos los días.	8 (8,1)	10 (10,1)	18 (18,2)	12 (12,1)	51 (51,5)
2. Si siento dificultad para respirar, descanso.	68 (68,7)	2 (2,0)	5 (5,1)	7 (7,1)	17 (17,2)
3. Si mi dificultad para respirar aumenta, me comunico con mi médico o con la enfermera (o con algún servicio de salud).	62 (62,6)	7 (7,1)	4 (4,0)	10 (10,1)	16 (16,2)
4. Si mis pies o piernas se hinchan más de lo habitual, me comunico con mi médico o con la enfermera (o con algún servicio de salud).	69 (69,7)	3 (3,0)	1 (1,0)	3 (3,0)	23 (23,2)
5. Si aumento 2 kg en una semana, me comunico con mi médico o con la enfermera (o con algún servicio de salud).	40 (40,4)	7 (7,1)	3 (3,0)	7 (7,1)	42 (42,4)
6. Limito la cantidad de líquidos que bebo (no más de 1,5 a 2 L al día).	29 (29,3)	7 (7,1)	7 (7,1)	3 (3,0)	53 (53,5)
7. Descanso durante el día.	79 (79,8)	5 (5,1)	8 (8,1)	1 (1,0)	6 (6,1)
8. Si mi cansancio aumenta, me comunico con mi médico o con la enfermera (o con algún servicio de salud).	75 (75,8)	7 (7,1)	5 (5,1)	2 (2,0)	10 (10,1)
9. Sigo una dieta baja en sal.	76 (76,8)	5 (5,1)	7 (7,1)	3 (3,0)	8 (8,1)
10. Tomo mi medicamento según la receta.	83 (83,8)	6 (6,1)	5 (5,1)	2 (2,0)	3 (3,0)
11. Me vacuno contra la gripe todos los años.	57 (57,6)	7 (7,1)	3 (3,0)	5 (5,1)	27 (27,3)
12. Hago ejercicio con regularidad.	26 (26,3)	10 (10,1)	8 (8,1)	8 (8,1)	47 (47,5)

La Tabla 3 presenta la matriz de correlación entre los puntajes de fatiga (DEFS y DUFS), autocuidado y variables sociodemográficas y clínicas. Se observó una correlación negativa significativa de magnitud baja entre la edad y la fatiga al esfuerzo (rho = −0,279; p < 0,01) y con la fatiga sustancial (rho = −0,274; p < 0,01). Hubo una correlación positiva significativa de magnitud moderada entre las variables NYHA y la fatiga de esfuerzo (rho = 0,501; p < 0,001) y de magnitud débil entre NYHA y la fatiga sustancial (rho = 0,251; p < 0,05). La duración del tratamiento presentó una correlación positiva débil con la edad (rho = 0,272; p < 0,01) y, por último, el número de medicamentos mostró una correlación positiva moderada con el número de comorbilidades (rho = 0,337; p < 0,01). En este estudio, no se encontró una correlación significativa entre la fatiga (DEFES y DUFS) y el autocuidado (p > 0,05).

**Tabla 3:** Matriz de correlaciones entre la fatiga, el autocuidado, las variables sociodemográficas y clínicas – João Pessoa, PB, Brasil, 2024.

Variables	1	2	3	4	5	6	7	8	9
1. Autocuidado	–	–	–	–	–	–	–	–	–
2. DEFS	0,065	–	–	–	–	–	–	–	–
3. DUFS	0,118	–	–	–	–	–	–	–	–
4. Edad	-0,003	-0,279**	-0,274**	–	–	–	–	–	–
5. NYHA	0,028	0,501***	0,251*	0,017	–	–	–	–	–
6. Tiempo de tratamiento	-0,074	-0,028	-0,049	0,272**	0,039	–	–	–	–
7. FEVI	-0,021	-0,001	-0,090	0,158	-0,090	0,151	–	–	–
8. Número de comorbilidades	0,058	-0,020	0,046	0,199	0,157	0,123	0,042	–	–
9. Número de medicamentos	-0,021	0,167	0,102	0,159	0,129	0,121	-0,122	0,337**	–

Abreviaturas: DUFS – *Dutch Fatigue Scale*; DEFS – *Dutch Exertion Fatigue Scale*; NYHA – *New York Heart Association*; FEVI – *Fracción de eyección del ventrículo izquierdo*.

*p < 0,05,

**p < 0,01,

***p < 0,001.

## DISCUSIÓN

En lo que respecta a las variables clínicas, los hallazgos concuerdan con los resultados de un estudio multicéntrico realizado en Francia, España, el Reino Unido, Alemania e Italia, que identificó una prevalencia similar al perfil clínico observado en esta investigación^(12)^.

Los hallazgos de este estudio pusieron de manifiesto una elevada prevalencia de fatiga sustancial ante el esfuerzo entre los participantes. Los resultados concuerdan con la literatura, la cual señala que alrededor del 60‒70% de las personas con IC reportan niveles moderados a elevados de fatiga, lo que pone de manifiesto no solo la frecuencia de este síntoma, sino también su persistencia a lo largo del curso de la enfermedad^(5)^. Además, la investigación señala que el aumento de la fatiga está asociado con el empeoramiento de la clase funcional, lo que sugiere un impacto directo en la capacidad funcional y en la tolerancia al esfuerzo^(13)^.

En un estudio realizado en São Paulo, los puntajes totales promedio de la DUFS y la DEFS fueron superiores a los observados en el presente estudio^(13)^. Esta diferencia puede estar relacionada con la gravedad de la IC o con las características clínicas de la muestra, lo que sugiere que los pacientes podrían encontrarse en etapas menos avanzadas de la enfermedad o con un control más eficaz de los síntomas.

En el contexto de la IC, la combinación de disfunción sistólica y diastólica compromete la capacidad de aumentar el gasto cardíaco. Como consecuencia, se instala un cuadro de fatiga persistente, en el que los pacientes agotados tienden a posponer u omitir prácticas de autocuidado, lo que agrava los síntomas, refuerza la apatía y aumenta la percepción de incapacidad para manejar su propio tratamiento. Esta condición repercute directamente en la dificultad para cumplir con actividades esenciales, como el control del peso corporal, la preparación de comidas equilibradas y el uso correcto de la medicación^(14)^.

Dichas evidencias indican que la fatiga no es solo una consecuencia de la enfermedad, sino también un factor que compromete el autocuidado, lo que da lugar a un círculo vicioso con impacto negativo sobre el pronóstico. Las intervenciones educativas sobre alimentación saludable, la práctica regular de actividad física moderada, la adherencia a la medicación y la conservación de la energía —incluyendo la planificación de las actividades diarias, la alternancia entre actividad y reposo, las técnicas de relajación, la movilización de la red de apoyo y la adaptación del entorno— han sido empleadas por el personal de enfermería en el manejo de la fatiga, contribuyendo al seguimiento a largo plazo, la prevención de complicaciones y a resultados clínicos favorables en pacientes con IC^(15,16,17)^. Por lo tanto, resulta oportuna la implementación de intervenciones de enfermería culturalmente adecuadas e individualizadas como componente esencial del manejo terapéutico de la fatiga.

Los resultados demostraron que los participantes de este estudio presentaron puntuaciones de autocuidado superiores a las observadas en un estudio nacional^(18)^ y en un estudio español^(19)^, lo que indica un desempeño menos favorable en las prácticas de autocuidado en comparación con los hallazgos de estas investigaciones. Dichas diferencias pueden estar asociadas a factores socioculturales, que influyen en la forma en que las personas perciben, comprenden y practican el autocuidado, lo que puede repercutir en el control de la condición de salud y, en consecuencia, en la calidad de vida^(4)^.

En cuanto a la frecuencia de los comportamientos de autocuidado, se observó que pesarse diariamente, informar sobre el aumento de peso inesperado y practicar ejercicio físico regularmente presentaron una mayor discrepancia entre los participantes. Estos resultados se asemejan a los de una investigación realizada en Suiza^(20)^. Además, se constató que los pacientes presentaban un bajo control de la ingesta diaria de líquidos, en consonancia con un estudio realizado en Japón^(21)^. Dichos resultados afectan el mantenimiento de la estabilidad clínica y el manejo de la enfermedad, lo que puede retrasar el reconocimiento temprano de la descompensación clínica para la toma de decisiones^(22)^.

Por otro lado, se identificó una mayor concordancia en comportamientos positivos de autocuidado en relación con el monitoreo y la notificación a un profesional de la salud de síntomas como dificultad para respirar, cansancio y edema, descanso durante el día, restricción del consumo de sal en la dieta, el uso regular de los medicamentos y la vacuna contra la gripe al día, que se asemejan a las variables encontradas en una investigación realizada en Eslovenia, cuyos resultados destacaron que el 94% de los participantes presentaba una buena adherencia a la medicación y el 86% restringía el consumo excesivo de sodio, lo que ayuda al paciente a lidiar positivamente con la IC y a mantener la enfermedad bajo control^(23)^.

Este estudio evidenció correlaciones significativas entre las variables sociodemográficas, clínicas y los índices de fatiga en pacientes con IC. Se observó una correlación negativa entre la edad y la fatiga por esfuerzo, lo que indica que los participantes más jóvenes referían una mayor sensación de cansancio durante las actividades cotidianas. Una posible explicación es que los adultos más jóvenes tienden a experimentar un mayor impacto funcional y psicológico ante las limitaciones que impone la enfermedad, lo que puede intensificar la percepción de fatiga. Se observó una correlación negativa entre la edad y la fatiga sustancial, lo que indica que los individuos más jóvenes presentaron una mayor intensidad de fatiga generalizada. Este hallazgo puede estar relacionado con la percepción individual respecto al reconocimiento de los síntomas de la insuficiencia cardíaca. Por el contrario, los pacientes de edad avanzada pueden tener más dificultades para reconocer estos síntomas, ya que los cambios derivados del propio proceso de envejecimiento reducen la reserva funcional y pueden contribuir al aumento de la fatiga, sobre todo cuando la IC se presenta junto con otras enfermedades crónicas^(24)^.

Además, se observó una correlación positiva entre la clase funcional (NYHA) y la fatiga al esfuerzo, lo que indica que, cuanto peor es la clasificación funcional, mayor es la limitación durante las actividades. De manera similar a este hallazgo, una investigación realizada en Suecia demostró, mediante los instrumentos RAND 36-*Item Health Survey* 1.0 (RAND- 36v1) y el *Work Productivity and Activity Impairment questionnaire* (WPAI), que el nivel de fatiga tiende a aumentar cuanto peor es la clasificación NYHA en el paciente con IC^(25)^.

Se identificó que, cuanto mayor es la edad del paciente, mayor es la duración del tratamiento, lo que indica que los pacientes de mayor edad tienden a permanecer en seguimiento durante períodos más prolongados. También se observó una correlación positiva entre el número de comorbilidades y la cantidad de medicamentos utilizados, lo que evidencia que, cuanto mayor es el número de afecciones clínicas asociadas, mayor tiende a ser el volumen de fármacos recetados. Se encontraron resultados similares en una investigación japonesa, que asoció la polifarmacia con un mayor número de comorbilidades, edad avanzada y peor pronóstico en pacientes con IC^(26)^.

Estos resultados refuerzan la necesidad de que el personal de enfermería planifique una atención que favorezca el uso terapéutico adecuado de los medicamentos, lo que incluye la identificación de vulnerabilidades, la prevención del uso indiscriminado de fármacos, estrategias para promover la adherencia al tratamiento, la participación de la familia en el proceso terapéutico y la garantía de una transición de cuidados segura y eficaz.

En esta investigación no se obtuvieron correlaciones significativas entre la fatiga y el autocuidado. Sin embargo, la literatura registra hallazgos distintos, que demuestran una asociación negativa entre estos parámetros cuando se evalúan mediante instrumentos como el *Self-Care of Heart Failure Index version* (SCHFI), lo que sugiere que las personas con mayor fatiga tienden a sentirse menos seguras para llevar a cabo comportamientos de autocuidado, lo cual repercute negativamente en la percepción de la salud y en el control de la enfermedad^(27)^.

Resultados recientes de un estudio japonés muestran que, aunque la fatiga no tiene un efecto directo sobre el autocuidado, esta relación está mediada por el control percibido. Por lo tanto, las intervenciones enfocadas en mejorar la percepción de control pueden influir positivamente en el manejo de los síntomas de la fatiga^(28)^. Además, la literatura señala que una mejor rutina de autorregulación ayuda a reconocer y manejar los síntomas físicos, pero que, dependiendo del nivel de fatiga, el comportamiento de autocuidado puede verse afectado^(29)^.

Este estudio presenta algunas limitaciones: el diseño transversal no permite establecer relaciones de causa y efecto. La recolección de datos en un solo centro restringe los resultados, y la medición de las variables mediante autoinforme está sujeta a sesgos de memoria y deseabilidad social. Además, la exclusión de pacientes en clase IV de la NYHA y la magnitud de las correlaciones observadas deben evaluarse con cautela al generalizar los hallazgos a la práctica clínica. En este sentido, se recomienda el desarrollo de estudios longitudinales, realizados en diferentes contextos del panorama brasileño y con muestras más amplias, para profundizar en la comprensión del impacto de la fatiga en el autocuidado de esta población.

En el ámbito de la práctica clínica multiprofesional, este estudio ofrece evidencia que puede contribuir al desarrollo de intervenciones longitudinales en la atención a personas con IC, especialmente aquellas orientadas al monitoreo sistemático de los síntomas y al fortalecimiento del autocuidado. Cabe destacar el papel central de la enfermería en este proceso, dada su actuación continua, cercana y educativa, que favorece la implementación de estrategias para la conservación de la energía, el manejo de la fatiga, la promoción de la adherencia terapéutica y la capacitación del paciente y su familia para el autocuidado. La adopción de estas medidas es fundamental para mejorar el seguimiento clínico, favorecer la estabilidad del estado de salud y, en consecuencia, mejorar los resultados clínicos y la calidad de vida.

## CONCLUSIÓN

En este estudio, la fatiga no se asoció con el comportamiento de autocuidado. Sin embargo, se observó una asociación significativa entre la edad y la fatiga de esfuerzo, la fatiga sustancial y la duración del tratamiento. La clase funcional de la NYHA también se mostró asociada a la fatiga de esfuerzo y a la fatiga sustancial.

Además, se constató una asociación positiva entre el número de comorbilidades y la polifarmacia en pacientes con insuficiencia cardíaca, un aspecto que puede influir directamente en el comportamiento de autocuidado. Estos hallazgos refuerzan la necesidad de implementar planes de atención orientados al control de los síntomas, con el fin de retrasar la progresión de la enfermedad, optimizar la gestión del autocuidado y promover una mejor calidad de vida.

## Data Availability

Todo el conjunto de datos que respalda los resultados de este estudio se publicó en el propio artículo.
